# Supplementary material for: Modeling the effects of perisaccadic attention on gaze statistics during scene viewing
Source: Commun Biol. 2020 Dec 1;3:727. doi: 10.1038/s42003-020-01429-8 (PMC7708631; doi:10.1038/s42003-020-01429-8)
Supplement: Supplementary file 1 — Supplementary Information [file 42003_2020_1429_MOESM1_ESM.pdf]

Supplementary Materials

This file includes:

- Figure S1 (Sample images from the experiments)
- Figure S2 (alternative visualization of the model phases)
- Figure S3 (Model performance on further statistics)
- Figure S4 (Correlations of empirical and simulated data)
- Figure S5 (Recovery Analysis)
- Figure S7 (Examples for subject-specific posteriors)
- Figure S6 (Estimated posteriors for model parameters)
- Table S1 (Fixed model parameters)
- Table S2 (Individual point estimates, baseline model)
- Table S3 (Individual point estimates, extended model)
- Supplementary Methods 1 (Example images used in the experiment)
- Supplementary Methods 2 (Model Phases)
- Supplementary Methods 3 (Fixed model parameters)
- Supplementary Note 1 (Results on systematic tendencies)
- Supplementary Note 2 (Results on Individual Differences)
- Supplementary Note 3 (Parameter recovery analysis)
- Supplementary Note 4 (Detailed results on parameter estimation)
- Supplementary Note 5 (Individual parameter estimates)

Supplementary Methods 1: Example images used in the experiment

The images used as stimuli in this study represent a subset of the Potsdam Corpus on Spatial Frequency Search in Natural Scenes[1]. Examples are given in Figure S1.

Supplementary Methods 2: Model Phases

In Figure S2 we show an alternative visualization of the temporal progression in the model.

Supplementary Methods 3: Fixed model parameters

In addition to the results on estimated model parameters we report the fixed model parameters in Table S1.

Supplementary Note 1: Results on systematic tendencies

In addition to the measures of scan path statistics reported in the *Results* we also investigated model performance with respect to two further statistics. First, as a measure of how fixations spread over an image over time we investigated the *mean lag distance*, defined as the distance between two fixations, separated by x other fixations (Fig. S3A). Empirical data indicate that the distance between fixation n and $n+x$ separate quickly for 3 to 5 fixations before reaching peak distance and returning to chance-level distance. We interpret this overshoot-type behavior as an indication of inhibitory tagging as one of the key driving mechanisms during scene exploration. The general tendency is present in the baseline model. While the extended model improves the fit to experimental data, the overshoot in the distance is not present. From this result, we might conclude that the inhibitor component is currently too weak in both mathematical models.

Second, an important systematic bias in eye movements is the central fixation tendency. Specifically, the first fixation in a scan path tends to be closer to center of the scene than subsequent fixations[2–5]. Figure S3C shows the distance to the image center over fixations. We added a center bias to the model by initializing the model in the attention stream with a centered

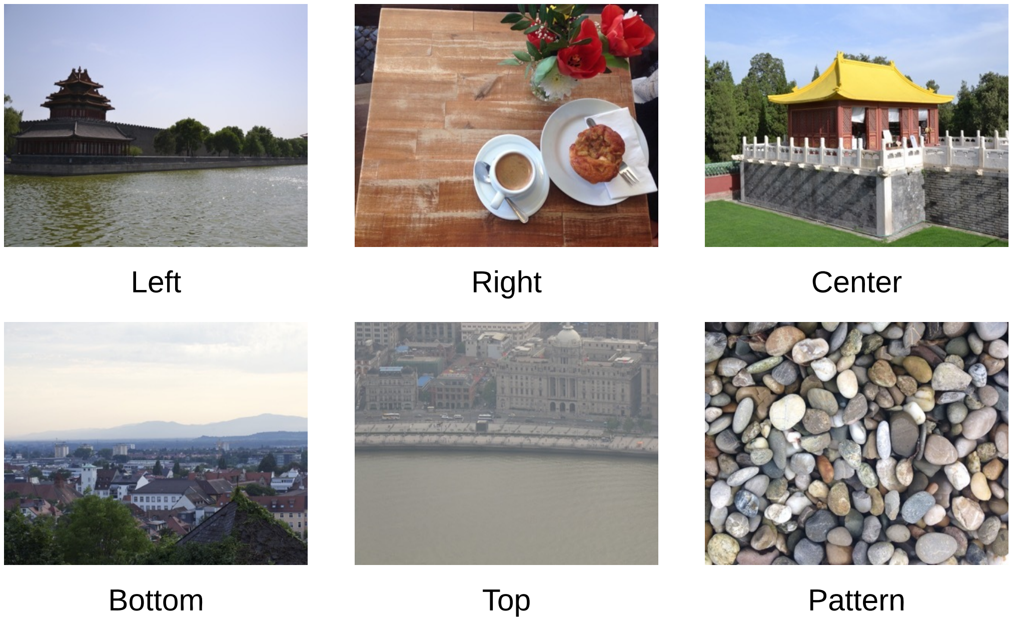

Figure S1. Six sample images from the scene viewing corpus. The first images are representative for categories of left, right, central, bottom, and top focus. The bottom right image provides an example for a natural pattern.

Gaussian activation map[4]. The characteristic dip on the second fixation (i.e., the first freely chosen fixation) is reproduced exactly by the new model.

Furthermore, the central fixation tendency is stronger when the initial saccade latency was shorter than on average[4]. Here we analyzed the dependence of the central fixation bias on the initial saccade latency (Figure S3B). Note that this analysis includes the full data set (i.e., the analysis is not limited to the test data) to produce more stable results. As a result, a larger latency before the first saccade is systematically related to a reduced central fixation bias compared to shorter fixations. The changes to the model improve the dynamic dependency of this measure.

Supplementary Note 2: Individual Differences

Using separate by-participant estimates, it is possible to examine how well the fitted model parameters capture inter-individual differences. For this analysis we compared the subject-specific experimental data to data simulated with the subject's set of parameters. We expect that subjects with high expression of a statistic should also show a higher degree of that statistic in simulated data. the specific model parameters fitted for each participant show good agreement between experimental and simulated data, and explain inter-individual differences, which we quantified by the corresponding correlation coefficient.

In Figure 3 we show the correlation of mean saccade amplitude in experimental and simulated data. Correspondingly, Figure S4A shows the correlations for the mean lag distance metric. We chose the distance at fixation 5 as our correlation measure, which on average is the point of peak distance in the experimental data. Both saccade amplitude and inhibition show good agreement between experimental and simulated data, suggesting that the model parameters capture the inter-individual differences adequately. The correlation is particularly interesting given that the mean lag distance peak is not strongly present in the simulated data.

Mean saccade amplitude (Fig. 3C) is closely related to the activation stream and lag distance to the inhibition stream. Thus, as the two most fundamental mechanisms in the model, the fact that they are well-represented in the individual model fits lends support to our model.

In Figure S4 B and C we show correlations between empirical and simulated data concerning the amount of forward and return saccades. These metrics represent the two most important new model components. The values we compare are the number of fixations per square degree that fall within a defined window for each peak. While there is more variation between subjects in the case of forward saccades, the magnitude of each tendency is well-represented by the model fit. The positive correlation of these measures shows that the model extension capture the relevant aspects in the data and account for inter-individual differences.

In general it is important to note that interindividual differences play a large role in eye movement behavior, particularly also concerning different participant groups such as children and/or patients[6,7]. In future work our new by-subject estimation

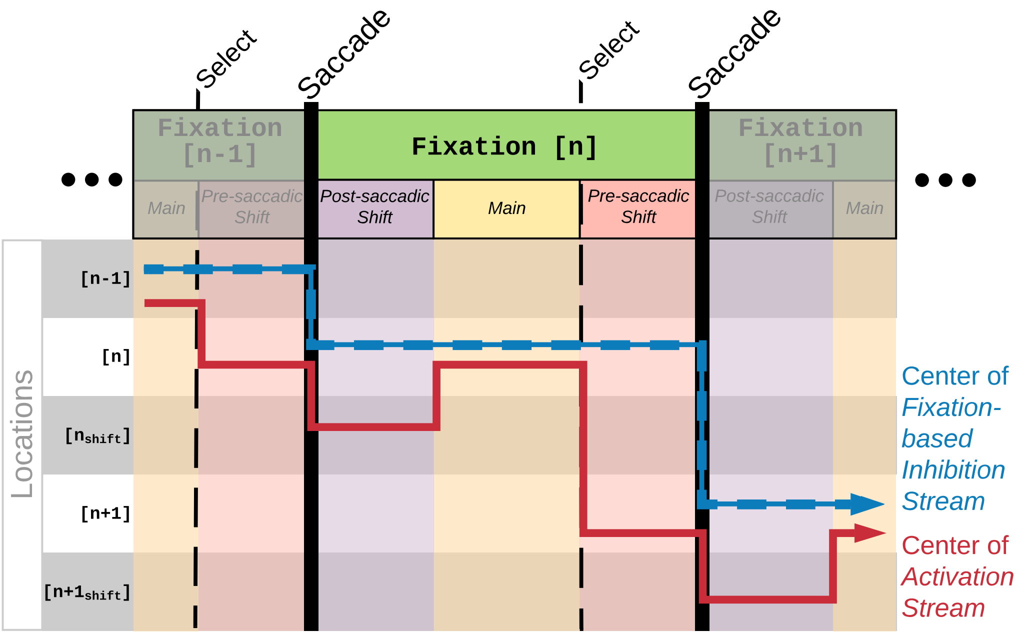

Figure S2. Fixation-phases of the activation and inhibition streams in the extended SceneWalk Model. The blue line shows the location around which the fixation-based inhibition stream's Gaussian aperture is centered. The red line represents the center of the activation stream. During each fixation, the model implements three phases. While the inhibition stream remains on the fixated location, the center of the activation stream shifts around the time of each saccade.

procedure could be applied to corresponding data.

Supplementary Note 3: Parameter recovery analysis

As an indicator for the reliability of our numerical simulations and, in particular, of our statistical inference, we investigated the parameter inference on simulated data with known parameters. We simulated data using parameter point estimates for one representative participant. Simulated data were then fed back to parameter inference, as outlined above (see *Methods*). In Figure S5 we report the posteriors over parameters in comparison to the true numerical values (χ was not included in this analysis). Thus, the combination of model simulations and the estimation procedures can recover values from the experimental data reliably. These analyses strengthen the credibility, stability, and robustness of our mathematical modeling approach.

Supplementary Note 4: Detailed results on parameter estimation

Based on the statistical methods described in the previous section, we obtained the parameter point estimates reported in Table 2 averaged across participants (see Tables S2 and S3).

As the parameter estimation was conducted in a fully Bayesian framework, we have also access to the full posterior likelihood distribution of each parameter. In Fig. S6 we show the marginal posterior distributions of each parameter of the model for all subjects. The marginal posterior distributions can serve as a tool to understand how well the parameters constrain the data.

For each parameter, we link the interpretation of the marginal posteriors to the function of the corresponding parameter in the model. The speed of the decay of the attention stream ω_A controls the duration of the memory of the process for allocation to past target locations. The numerical value $\omega_A = 10.12$ is indicates a half life of 70 ms of the previous map's influence (i.e., $\exp(-10.12 \cdot 0.070) = 0.49$). The Gaussian of the attention and inhibition streams are specified by parameters $\sigma_A = 7.3$ and $\sigma_F = 6.9$, resp., which are corresponding to standard deviation parameters of the Gaussian function in units of degrees of visual angle. The marginal posteriors of both parameters are largely overlapping. The exponent $\gamma \approx 1$ indicates that the weighting of the corresponding activation maps is negligible. Finally, the shift parameter η is clearly smaller than one, as expected.

There are two indicators lend support to the stability of our parameter estimations using the baseline SceneWalk model and the extended model in combination with the DREAM method. Firstly, the three chains which we ran for each subject resemble each other quite closely. Also, while the parameter estimates vary between participants, in most cases they do not differ dramatically (see Fig. S7). Secondly, we conducted recovery analyses of the estimated parameters, where a parameter

Parameter	Baseline SceneWalk	Extended Model
ω_A/ω_F	10	10
CF	0.3	0.3
τ_{pre}	–	0.05
τ_{post}	–	0.1
ν	–	2
σ_{post}	–	2
ω_{CB}	–	1.5
σ_{CBx}	–	4
σ_{CBy}	–	3
ω_A/ω_{FoR}	–	10

Table S1. Fixed model parameters for baseline and extended model.

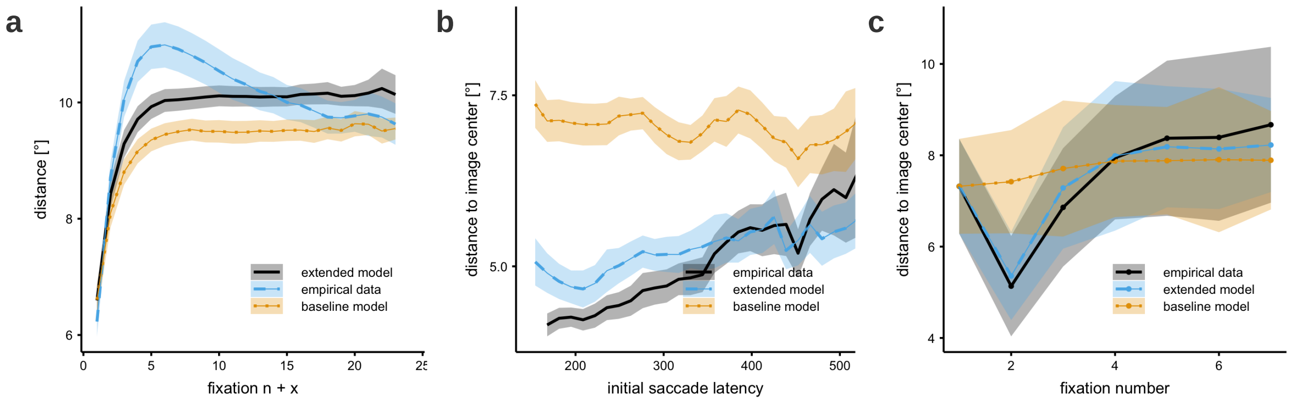

Figure S3. Model performance on additional statistics. (A) Mean distance between a fixation and subsequent fixations in experimental and simulated data. (B) Modulation of the the first fixation's mean distance to the image center by initial saccade latency. (C) The distance to the image center of each fixation in the sequence. The empirical tendency to move close to the center at fixation 2 is well-replicated by the extended model.

estimation is run on simulated data. The dream algorithm was able to identify the parameter values from the simulated data (see parameter recovery).

Supplementary Note 5: Individual parameter estimates

Each subject's data was individually fitted to both the baseline model and the extended model. In Table S2 we report the results of parameter estimation of the baseline model (SceneWalk). Table S3 gives the estimated parameters for the extended model. For each estimated parameter we computed the point estimate and the corresponding 50% credibility interval.

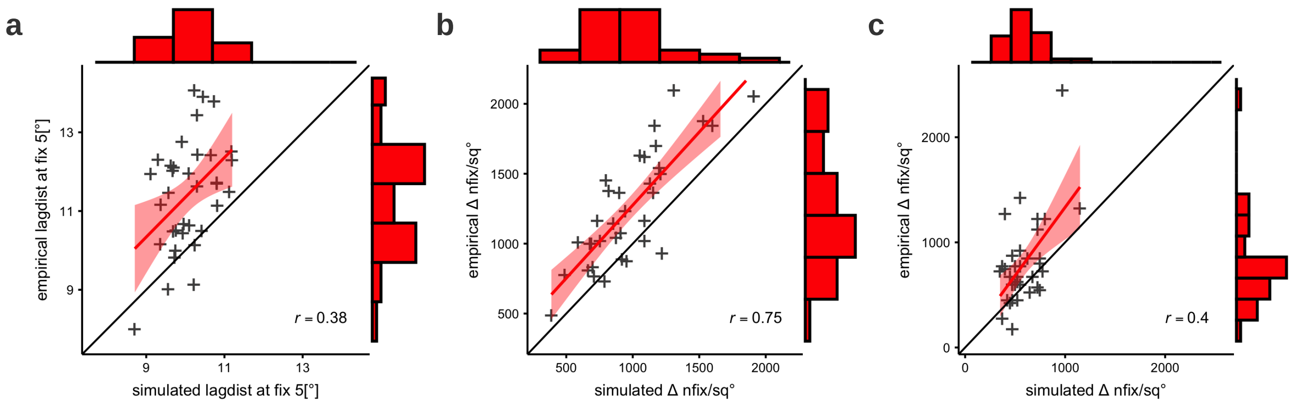

Figure S4. **Correlations between experimental and simulated data across participants.** (A) Mean lag distance at fixation 5. (B) Number of fixations per square degree that land within the area expected to contain forward saccades. (C) Number of fixations per square degree that land in the area expected to contain return saccades.

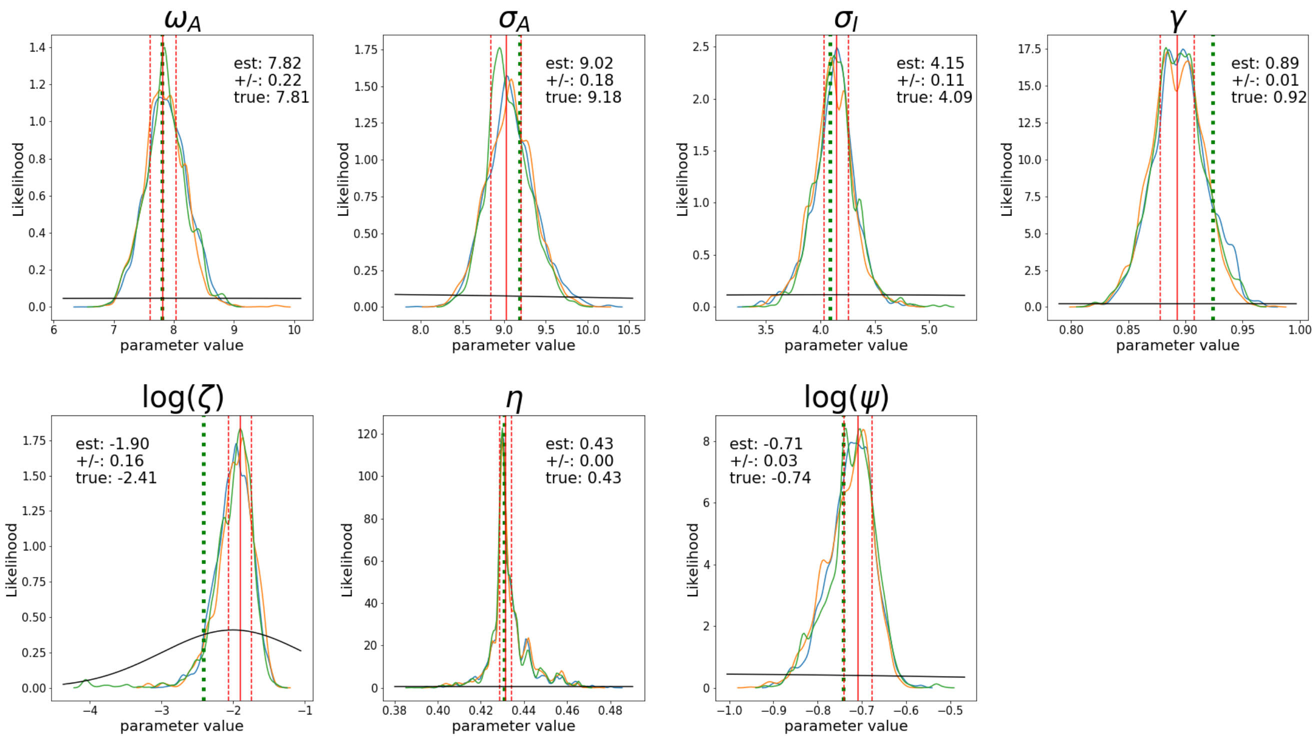

Figure S5. **Model parameter recovery analysis.** Panels report posteriors for estimated model parameters obtained from simulated data. The three curves represent three estimation chains of the DREAM algorithm. The green, dotted line is the parameter value from which data was generated. The red lines show the recovered parameter values: the maximum posteriori value (solid line) and the 50% credibility interval (dotted line). Black lines show the prior.

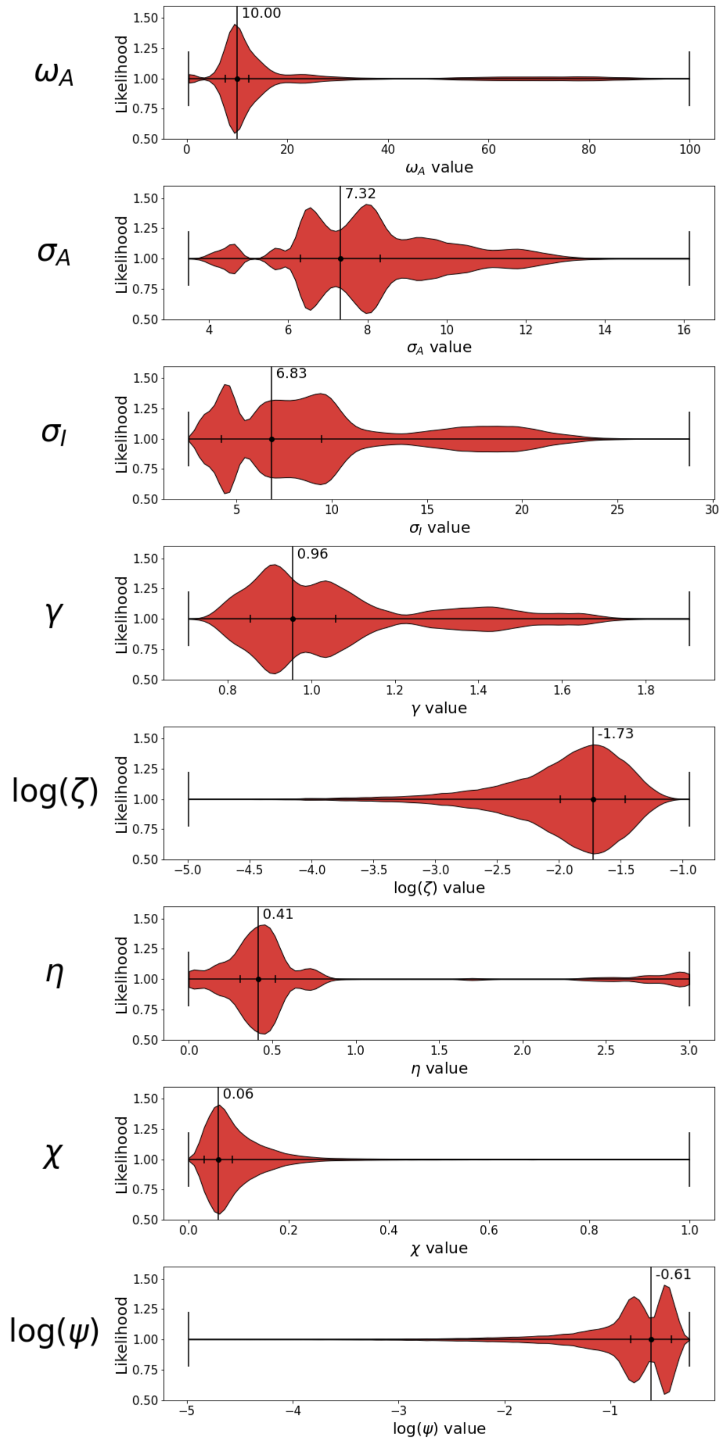

Figure S6. **Marginal posteriors of the estimated model parameters as calculated by fitting the model to the training data using PyDream.**

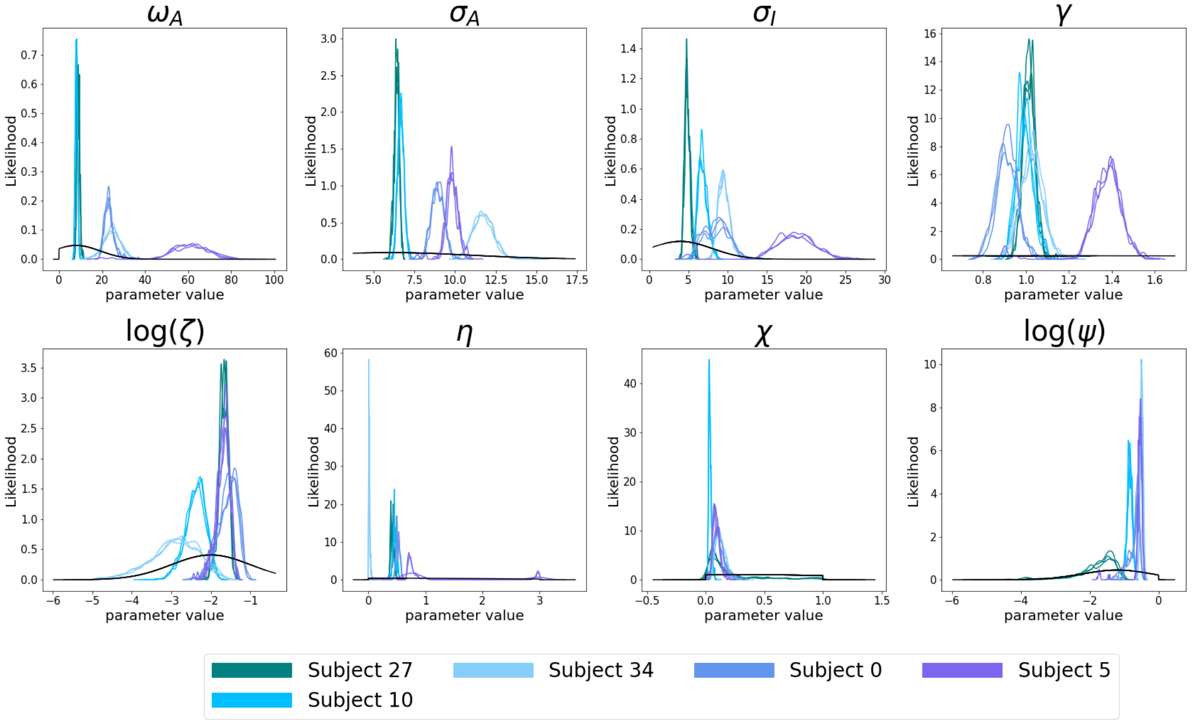

Figure S7. Posterior density of individuals and chains of the model fit using DREAM. The different colors show chains belonging to one subject. The black line is the prior. The consistency of the chains within each subject indicates that we achieved a reliable fit. All chains are markedly different from the posterior, allowing us to update our beliefs.

Supplementary References

References

1. Rothkegel, L., Schütt, H., Trukenbrod, H. A., Wichmann, F. & Engbert, R. Potsdam Scene Viewing Corpus, DOI: 10.17605/OSF.IO/N3BYQ (2019).
2. Tatler, B. W. The central fixation bias in scene viewing: Selecting an optimal viewing position independently of motor biases and image feature distributions. *J. Vis.* **7**, 4:1–17 (2007).
3. Bindemann, M. Scene and screen center bias early eye movements in scene viewing. *Vis. Res.* **50**, 2577–2587 (2010).
4. Rothkegel, L. O. M., Trukenbrod, H. A., Schütt, H. H., Wichmann, F. A. & Engbert, R. Temporal evolution of the central fixation bias in scene viewing. *J. Vis.* **17**, 3, DOI: 10.1167/17.13.3 (2017).
5. van Renswoude, D. R., van den Berg, L., Raijmakers, M. E. J. & Visser, I. Infants' center bias in free viewing of real-world scenes. *Vis. Res.* **154**, 44–53, DOI: 10.1016/j.visres.2018.10.003 (2019).
6. Le Meur, O. *et al.* Visual attention saccadic models learn to emulate gaze patterns from childhood to adulthood. *IEEE Transactions on Image Process.* **26**, 4777–4789, DOI: 10.1109/tip.2017.2722238 (2017).
7. Helo, A., Pannasch, S., Sirri, L. & Rämä, P. The maturation of eye movement behavior: Scene viewing characteristics in children and adults. *Vis. Res.* **103**, 83–91, DOI: 10.1016/j.visres.2014.08.006 (2014).

Subject	γ	γ +/-	ω_A	ω_A +/-	σ_A	σ_A +/-	σ_F	σ_F +/-	$log(\zeta)$	$log(\zeta)$ +/-
0	0.927	0.025	24.368	1.702	7.551	0.141	5.566	0.264	-1.053	0.041
1	0.911	0.026	12.819	0.825	7.919	0.134	3.144	0.103	-1.172	0.057
2	1.082	0.023	17.484	1.154	6.406	0.101	5.111	0.208	-1.162	0.044
3	0.752	0.019	15.499	0.919	6.605	0.101	3.760	0.212	-1.402	0.072
4	1.409	0.030	44.364	2.301	9.069	0.183	0.061	0.014	-1.974	0.270
5	1.070	0.020	12.667	0.921	8.067	0.175	4.651	0.232	-1.343	0.058
6	0.963	0.020	14.522	0.791	5.137	0.085	3.333	0.181	-1.178	0.047
7	1.091	0.026	11.743	0.660	4.054	0.064	3.744	0.128	-1.056	0.031
8	0.741	0.014	18.658	1.272	5.595	0.090	5.228	0.178	-1.104	0.045
9	0.990	0.019	10.869	0.631	5.632	0.072	3.728	0.098	-1.697	0.077
10	0.960	0.027	12.875	0.925	6.161	0.097	5.844	0.224	-1.651	0.083
11	1.141	0.027	21.191	1.683	8.478	0.145	6.282	0.267	-1.091	0.041
12	0.935	0.021	14.712	1.112	4.923	0.090	3.760	0.228	-1.093	0.040
13	0.867	0.020	13.251	0.840	4.893	0.078	4.288	0.154	-1.366	0.050
14	0.810	0.018	15.372	0.929	7.214	0.109	4.275	0.190	-1.608	0.098
15	1.245	0.026	16.038	1.688	7.659	0.159	8.140	0.803	-1.106	0.035
16	0.982	0.029	21.626	2.352	4.775	0.077	5.497	0.234	-1.028	0.033
17	0.913	0.018	13.101	0.996	7.977	0.136	5.982	0.255	-1.896	0.136
18	0.978	0.020	16.447	1.258	8.533	0.166	6.587	0.333	-1.225	0.051
19	0.917	0.018	12.971	0.879	6.828	0.120	4.256	0.164	-1.199	0.050
20	0.958	0.033	13.985	1.574	8.016	0.177	7.987	0.502	-1.040	0.041
21	0.943	0.014	8.820	0.802	6.920	0.136	6.137	0.483	-1.496	0.062
22	1.367	0.029	15.801	0.892	8.700	0.135	3.196	0.110	-0.971	0.035
23	0.952	0.038	13.998	1.466	6.886	0.153	16.378	1.585	-1.045	0.044
24	1.167	0.021	15.325	1.163	6.360	0.104	5.206	0.277	-1.266	0.044
25	1.067	0.018	16.058	1.094	7.538	0.116	5.264	0.250	-1.384	0.054
26	1.229	0.030	8.680	0.869	9.245	0.269	5.290	0.271	-1.213	0.079
27	0.859	0.018	18.938	1.356	4.884	0.098	4.240	0.210	-1.525	0.085
28	1.196	0.019	20.110	1.568	8.180	0.142	5.410	0.199	-1.170	0.043
29	1.463	0.036	13.326	2.919	10.551	0.243	16.203	1.534	-1.076	0.036
30	0.920	0.019	15.208	0.947	7.034	0.122	3.822	0.132	-1.201	0.051
31	0.231	0.011	43.633	3.538	2.939	0.091	3.158	0.179	-0.977	0.045
32	0.862	0.023	16.398	1.213	3.920	0.070	2.775	0.122	-1.294	0.048
33	1.067	0.024	14.702	1.038	5.711	0.076	3.825	0.155	-1.261	0.045
34	0.944	0.032	24.298	2.569	9.347	0.227	6.307	0.349	-0.898	0.043

Table S2. Estimated parameter values of the baseline SceneWalk model for all participants (see Table S1 for fixed parameters).

Subject	χ	χ +/-	η	η +/-	γ	γ +/-	ω_A	ω_A +/-	σ_A	σ_A +/-	σ_F	σ_F +/-	$log(\psi)$	$log(\psi)$ +/-	$log(\zeta)$	$log(\zeta)$ +/-
0	0.090	0.027	0.511	0.022	0.905	0.032	22.820	1.305	8.941	0.268	9.185	1.177	-0.611	0.067	-1.460	0.158
1	0.066	0.030	0.441	0.015	0.904	0.026	8.661	0.714	9.180	0.187	3.426	0.234	-0.958	0.285	-1.493	0.139
2	0.082	0.024	0.413	0.018	0.911	0.025	15.505	0.981	7.104	0.181	7.282	0.460	-0.758	0.099	-1.689	0.144
3	0.055	0.020	0.493	0.019	0.802	0.022	12.810	0.642	7.532	0.168	4.458	0.213	-1.083	0.103	-1.673	0.092
4	0.175	0.043	2.974	0.026	1.339	0.048	79.172	3.848	8.326	0.219	18.587	1.275	-0.445	0.027	-2.102	0.214
5	0.081	0.019	0.727	0.040	1.385	0.040	59.578	5.479	9.725	0.226	18.229	1.609	-0.539	0.034	-1.646	0.104
6	0.064	0.015	0.417	0.009	1.033	0.026	11.031	0.488	6.813	0.151	6.111	0.490	-0.836	0.067	-1.632	0.094
7	0.118	0.021	0.494	0.018	1.444	0.042	8.440	0.462	4.217	0.159	15.433	1.436	-0.349	0.025	-1.616	0.118
8	0.056	0.008	0.428	0.021	1.051	0.026	9.341	0.514	7.569	0.198	9.563	0.636	-0.471	0.027	-1.864	0.140
9	0.041	0.041	0.509	0.016	1.099	0.018	8.598	0.334	6.379	0.113	3.151	0.111	-1.969	0.304	-1.730	0.077
10	0.031	0.007	0.463	0.018	0.992	0.025	7.980	0.361	6.655	0.127	6.689	0.376	-0.827	0.047	-2.362	0.167
11	0.102	0.033	0.399	0.014	0.932	0.021	12.103	0.949	9.466	0.258	8.558	0.391	-0.874	0.053	-1.508	0.072
12	0.060	0.010	0.307	0.017	1.084	0.026	10.367	0.703	6.418	0.146	12.359	0.723	-0.451	0.018	-2.280	0.219
13	0.065	0.014	0.522	0.013	0.899	0.020	9.628	0.399	5.692	0.098	6.871	0.310	-0.793	0.043	-1.949	0.110
14	0.085	0.048	0.583	0.022	0.810	0.023	14.693	0.740	7.931	0.120	4.586	0.162	-1.327	0.198	-1.947	0.145
15	0.094	0.019	0.318	0.025	0.908	0.022	7.679	0.666	8.342	0.217	10.099	0.410	-0.688	0.026	-2.845	0.379
16	0.069	0.012	0.350	0.009	1.284	0.028	10.213	0.503	6.709	0.172	17.440	1.361	-0.513	0.025	-1.411	0.058
17	0.038	0.034	0.382	0.025	0.886	0.020	13.462	1.033	8.223	0.162	6.178	0.298	-1.446	0.249	-1.715	0.091
18	0.044	0.009	0.323	0.011	1.044	0.022	9.492	0.546	11.713	0.375	9.008	0.789	-0.656	0.038	-1.908	0.127
19	0.114	0.089	0.302	0.019	0.866	0.018	8.752	0.679	7.804	0.172	4.349	0.146	-1.486	0.304	-1.385	0.066
20	0.077	0.022	0.751	0.019	0.982	0.037	29.371	3.378	8.213	0.200	9.908	0.831	-0.729	0.055	-1.264	0.064
21	0.060	0.014	0.368	0.022	1.024	0.028	10.314	0.958	8.103	0.164	9.421	0.562	-0.810	0.045	-1.901	0.113
22	0.147	0.023	2.816	0.136	1.469	0.036	70.456	3.942	10.353	0.248	15.990	0.947	-0.442	0.021	-2.528	0.347
23	0.051	0.007	0.198	0.019	1.402	0.049	0.625	0.040	10.185	0.679	18.559	1.445	-0.426	0.021	-1.946	0.129
24	0.108	0.024	0.164	0.012	1.160	0.023	9.564	0.448	8.012	0.199	7.788	0.357	-0.756	0.036	-1.984	0.131
25	0.030	0.005	0.037	0.023	0.827	0.020	12.721	1.070	7.789	0.203	8.460	0.536	-0.816	0.039	-2.485	0.222
26	0.052	0.014	0.161	0.023	1.133	0.032	6.289	0.558	11.923	0.545	9.724	0.713	-0.742	0.042	-2.161	0.225
27	0.072	0.046	0.392	0.021	1.016	0.019	8.630	0.448	6.435	0.098	4.820	0.217	-1.505	0.253	-1.667	0.080
28	0.067	0.010	2.529	0.121	1.621	0.049	84.455	4.655	9.431	0.238	19.496	1.182	-0.374	0.015	-2.264	0.205
29	0.157	0.029	2.755	0.062	1.621	0.042	60.145	4.287	10.576	0.265	20.243	1.272	-0.507	0.019	-1.955	0.139
30	0.035	0.010	0.483	0.032	0.828	0.018	10.687	0.555	7.496	0.163	4.320	0.265	-1.129	0.092	-1.500	0.077
31	0.071	0.013	0.687	0.011	0.934	0.024	13.099	0.618	6.988	0.141	9.374	0.539	-0.524	0.024	-2.094	0.167
32	0.031	0.009	0.488	0.017	0.919	0.018	9.758	0.409	4.633	0.076	3.845	0.159	-1.017	0.080	-1.718	0.092
33	0.057	0.014	0.253	0.011	0.941	0.025	18.227	1.651	6.418	0.144	6.284	0.580	-0.811	0.076	-1.785	0.110
34	0.111	0.027	0.013	0.006	1.030	0.035	24.958	2.591	11.618	0.429	9.267	0.477	-0.486	0.028	-2.767	0.402

Table S3. Estimated parameter values of the extended SceneWalk model for all participants (see Table S1 for fixed parameters).
