# Supplementary material for: Modeling the effects of perisaccadic attention on gaze statistics during scene viewing
Source: Commun Biol. 2020 Dec 1;3:727. doi: 10.1038/s42003-020-01429-8 (PMC7708631; doi:10.1038/s42003-020-01429-8)
Supplement: Supplementary file 3 — Reporting Summary [file 42003_2020_1429_MOESM3_ESM.pdf]

nature research

Corresponding author(s): Lisa Schwetlick (lisa.schwetlick@uni-potsdam.de)

Last updated by author(s): 29.9.2020

Reporting Summary

Nature Research wishes to improve the reproducibility of the work that we publish. This form provides structure for consistency and transparency in reporting. For further information on Nature Research policies, see our Editorial Policies and the Editorial Policy Checklist.

Statistics

For all statistical analyses, confirm that the following items are present in the figure legend, table legend, main text, or Methods section.

n/a	Confirmed	
☐	☒	The exact sample size (*n*) for each experimental group/condition, given as a discrete number and unit of measurement
☐	☒	A statement on whether measurements were taken from distinct samples or whether the same sample was measured repeatedly
☐	☒	The statistical test(s) used AND whether they are one- or two-sided<br>*Only common tests should be described solely by name; describe more complex techniques in the Methods section.*
☐	☒	A description of all covariates tested
☐	☒	A description of any assumptions or corrections, such as tests of normality and adjustment for multiple comparisons
☐	☒	A full description of the statistical parameters including central tendency (e.g. means) or other basic estimates (e.g. regression coefficient) AND variation (e.g. standard deviation) or associated estimates of uncertainty (e.g. confidence intervals)
☒	☐	For null hypothesis testing, the test statistic (e.g. F, t, r) with confidence intervals, effect sizes, degrees of freedom and P value noted<br>*Give P values as exact values whenever suitable.*
☐	☒	For Bayesian analysis, information on the choice of priors and Markov chain Monte Carlo settings
☒	☐	For hierarchical and complex designs, identification of the appropriate level for tests and full reporting of outcomes
☒	☐	Estimates of effect sizes (e.g. Cohen's d, Pearson's r), indicating how they were calculated

Our web collection on statistics for biologists contains articles on many of the points above.

Software and code

Policy information about availability of computer code

Data collection	Data collection was not part of this work. The data used is available at osf.io/caqt2.
Data analysis	Source code used for numerical simulations, analyses, and plotting as well as other project-related files are made available (osf.io/qsx4w).

For manuscripts utilizing custom algorithms or software that are central to the research but not yet described in published literature, software must be made available to editors and reviewers. We strongly encourage code deposition in a community repository (e.g. GitHub). See the Nature Research guidelines for submitting code & software for further information.

Data

Policy information about availability of data

All manuscripts must include a data availability statement. This statement should provide the following information, where applicable:

- Accession codes, unique identifiers, or web links for publicly available datasets
- A list of figures that have associated raw data
- A description of any restrictions on data availability

The experimental data used in this study represent a subset of the Potsdam Corpus on Spatial Frequency Search in Natural Scenes67, which is publicly available via the Open Science Framework (osf.io/caqt2).

April 2020

Field-specific reporting

Please select the one below that is the best fit for your research. If you are not sure, read the appropriate sections before making your selection.

☐ Life sciences ☒ Behavioural & social sciences ☐ Ecological, evolutionary & environmental sciences

For a reference copy of the document with all sections, see nature.com/documents/nr-reporting-summary-flat.pdf

Behavioural & social sciences study design

All studies must disclose on these points even when the disclosure is negative.

Study description	This work develops a dynamical model of human eye movement behavior.
Research sample	The data were collected as part of a previous study and are freely available at the Open Science Framework (osf.io/caqt2). We used a subset of this data, namely experiment 3. This subset contains the eye movement recordings of 35 participants who viewed 30 images for 10 seconds each. 312, 267 fixations and saccades were detected for further analysis. For the parameter estimation we treated each subject as a separate dataset, and ran the estimation procedure for each individual.
Sampling strategy	N/A
Data collection	N/A
Timing	N/A
Data exclusions	Data point that were unusable due to eye blinks were removed from the sample.
Non-participation	N/A
Randomization	For the parameter estimation we used randomized training and test sets. Training sets contained 75% of the data.

Reporting for specific materials, systems and methods

We require information from authors about some types of materials, experimental systems and methods used in many studies. Here, indicate whether each material, system or method listed is relevant to your study. If you are not sure if a list item applies to your research, read the appropriate section before selecting a response.

Materials & experimental systems

n/a	Involved in the study
☒	☐ Antibodies
☒	☐ Eukaryotic cell lines
☒	☐ Palaeontology and archaeology
☒	☐ Animals and other organisms
☒	☐ Human research participants
☒	☐ Clinical data
☒	☐ Dual use research of concern

Methods

n/a	Involved in the study
☒	☐ ChIP-seq
☒	☐ Flow cytometry
☒	☐ MRI-based neuroimaging
